# A retrospective analysis of the effect of intraoperative opioid dose on cancer recurrence after non-small cell lung cancer resection

**DOI:** 10.1002/cam4.236

**Published:** 2014-04-02

**Authors:** Juan P Cata, Visesh Keerty, Dinesh Keerty, Lei Feng, Peter H Norman, Vijaya Gottumukkala, John R Mehran, Mitchel Engle

**Affiliations:** 1 Department of Anesthesiology and Perioperative Medicine, The University of Texas – MD Anderson Cancer CenterHouston, Texas; 2 Department of Internal Medicine, The Wright Center for Graduate Medical EducationScranton, Pennsylvania, USA; 3 Department of Biostatistics, The University of Texas – MD Anderson Cancer CenterHouston, Texas; 4 Department of Thoracic Surgery, The University of Texas – MD Anderson Cancer CenterHouston, Texas; 5 Department of Pain Medicine, The University of Texas – MD Anderson Cancer CenterHouston, Texas

**Keywords:** Neoplasms, opioids, recurrence, surgery

## Abstract

Preclinical studies have demonstrated that opioid receptor agonists increase the rate of non-small cell lung cancer (NSCLC) growth and metastasis. Following institutional review board approval, we retrieved data on 901 patients who underwent surgery for NSCLC at MD Anderson Cancer Center. Comprehensive demographics, intraoperative data, and recurrence-free survival (RFS) and overall survival (OS) at 3 and 5 years were obtained. Cox proportional analyses were conducted to assess the association between intraoperative opioid exposure and RFS and OS. The median intraoperative fentanyl equivalents dosage was 10.15 *μ*g/kg. The multivariate analysis by stage indicated that a trend toward significance for opioid consumption as a risk factor in stage I patients (*P* = 0.053). No effect was found on RFS for stage II or III patients. Alternatively, opioid consumption was a risk factor for OS for stage I patients (*P* = 0.036), whereas no effect was noted for stage II or III patients. Intraoperative opioid use is associated with decreased OS in stage I but not stage II–III NSCLC patients. Until randomized controlled studies explore this association further, opioids should continue to be a key component of balanced anesthesia.

## Introduction

Opioids are the most commonly used intraoperative analgesics. Immunosuppression with opioids is commonly reported as a reduction in the natural killer cell activity against cancer cells and a potential contributor to an increase in metastasis 1. Importantly, opioids are also reported to directly enhance cancer growth through coactivation of the vascular endothelial growth factors (VEGF) 2,3. Furthermore, certain polymorphisms in the *μ*-opioid receptor (MOR) have been associated with different rate of survival in patients with breast and esophageal cancer 4,5. These laboratory and epidemiological observations warrant further clinical studies to evaluate the potential link between perioperative opioid use and recurrence-free survival (RFS) 6,7. To date, clinical studies evaluating the association between opioid-based anesthesia and regional analgesia techniques on cancer recurrence in breast, prostate, ovarian, and colorectal cancer are mixed and inconclusive 8. In the nonsurgical patient population with advanced cancers, there is evidence associating the use of opioids with poor prognosis 9,10. Interestingly, the use of the *μ*-receptor antagonist naltrexone along with radiotherapy appears to prolong survival in patients with brain tumors 11.

As most of the studies evaluating the effects of opioids on tumor growth come from experimental lung cancer models, we decided to test the hypothesis that high intraoperative doses of opioids would be associated with decreased recurrence and overall free survivals.

## Methods

### Patient selection

After approval from The University of Texas MD Anderson Cancer Center's institutional review board, we recorded information from 1154 patients with stage I–IIIa non-small cell lung cancer (NSCLC) who underwent surgery from January of 2004 through June of 2009. We included patients 18 years of age or older and we excluded diagnostic procedures such as endobronchial ultrasounds and those patients who had thoracic surgery for noncurative resections (*n* = 163).

**Figure 1:**
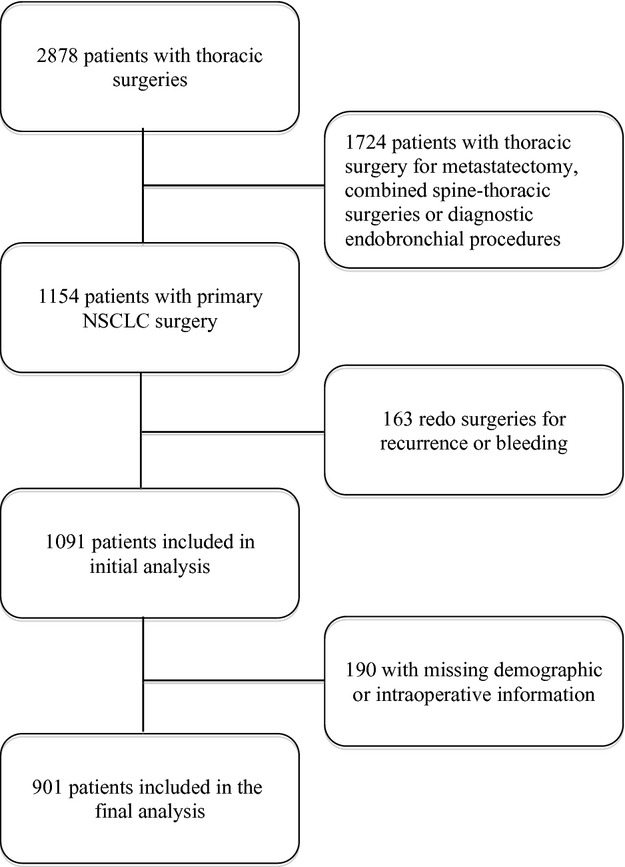
Flow diagram.

We collected the following data from MD Anderson Cancer Center electronic medical records and tumor registry. Patient with missing information were not included in the analysis (*n* = 190, Fig.1). The total amount of fentanyl equivalents received during surgery was exposure variable. The fentanyl equivalents conversion factors for 1 *μ*g fentanyl were as follows: sufentanil 0.1 *μ*g, remifentanil 1 *μ*g, and hydromorphone 10 *μ*g.

The a priori primary endpoint of this study was RFS, defined as the time in months from surgery to recurrence or death, whichever event occurred first. Overall survival (OS) was defined as the time in months from surgery to death from any cause.

### Statistical analysis

Summary statistics including mean, standard deviation, median, and range were recorded and calculated for continuous variables. Frequency counts and percentages for categorical variables were calculated. Fisher's exact test or chi-square test was used to evaluate the association between two categorical variables. Logarithmic transformation was used for the continuous variables such as fentanyl equivalents and duration of surgery in minutes. BLiP plots were generated for fentanyl equivalents in logarithmic scale for each of the categorical variables. Wilcoxon rank sum test or Kruskal–Wallis test was used to evaluate the difference in fentanyl equivalents in logarithmic scale between/among patient groups. Scatter plots were generated to evaluate the correlation between fentanyl equivalents and duration of surgery in minutes in logarithmic scale. Kaplan–Meier method was used for time-to-event analysis including RFS and OS. Median time-to-event in months with 95% confidence interval was calculated. The log-rank test was used to evaluate the difference in time-to-event endpoints between patient groups. Univariate Cox proportional hazards models were fitted to evaluate the effects of continuous variables on time-to-event outcomes. Multivariable Cox proportional hazards models were used for multivariate analysis to include important and significant covariates. Statistical software SAS 9.1.3 (SAS, Cary, NC) and S-Plus 8.0 (TIBCO Software Inc., Palo Alto, CA) were used for all the analyses.

## Results

We analyzed data from 901 patients (Fig.1). Of which, 425 were women (47.17%) and 476 men (52.83%). The median age of the study population was 66 years. Most patients had stage I cancer (54.06%) and an ASA physical status of 3 (84.9%). The most common surgical approach was an open thoracotomy (78.9%) and the most common procedure was a lobectomy (77.3%). These and other demographic characteristics are listed in Table1. Majority of the patients had combined balanced general-epidural anesthesia. Intraoperative analgesia was achieved with intravenous infusion or boluses of sufentanil, remifentanil, and/or fentanyl.

**Table 1:** Demographic characteristics by cancer stage.

Variable	Levels	Stage I	Stage II	Stage III	*P*-value
Age (years)	<65	198 (40.7%)	86 (46%)	109 (48.2%)	0.1345
	≥65	288 (59.3%)	101 (54%)	117 (51.8%)	
BMI	≤25	185 (38.1%)	62 (33.2%)	84 (37.2%)	0.4927
	>25	301 (61.9%)	125 (66.8%)	142 (62.8%)	
Gender	Female	254 (52.3%)	70( 37.4%)	101 (44.7%)	0.0017
	Male	232 (47.7%)	117 (62.6%)	125 (55.3%)	
ASA physical status	2	46 (9.5%)	18 (9.6%)	21 (9.3%)	0.8847
	3	410 (84.7%)	158 (84.5%)	196 (86.7%)	
	4	28 (5.8%)	11 (5.9%)	9 (4%)	
Fentanyl (*μ*g/kg)	<10.15	268 (55.1%)	83 (44.4%)	98 (43.4%)	0.0032
	≥10.15	218 (44.9%)	104 (55.6%)	128 (56.6%)	
Preoperative chemotherapy	N	439 (90.3%)	152 (81.3%)	123 (54.7%)	<0.0001
	Y	47 (9.7%)	35 (18.7%)	102 (45.3%)	
Preoperative radiation	N	479 (98.6%)	182 (97.3%)	204 (90.3%)	<0.0001
	Y	7 (1.4%)	5 (2.7%)	22 (9.7%)	
Postoperative chemotherapy	N	403 (83.1%)	101 (54%)	117 (51.8%)	<0.0001
	Y	82 (16.9%)	86 (46%)	109 (48.2%)	
Postoperative radiation	N	468 (96.5%)	164 (87.7%)	129 (57.3%)	<0.0001
	Y	17 (3.5%)	23 (12.3%)	96 (42.7%)	
Thoracoscopy	N	372 (76.5%)	177 (95.2%)	208 (92%)	<0.0001
	Y	114 (23.5%)	9 (4.8%)	18 (8%)	

**Figure 2:**
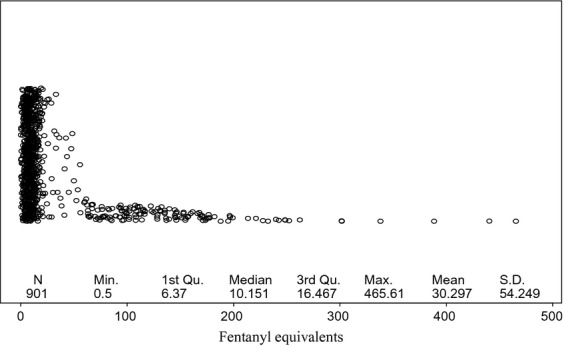
Blip plot for fentanyl equivalent use. The figure depicts the skewed distribution of fentanyl equivalents/kg consumption. The median amount of fentanyl equivalent use was 10.15 *μ*g/kg.

**Figure 3:**
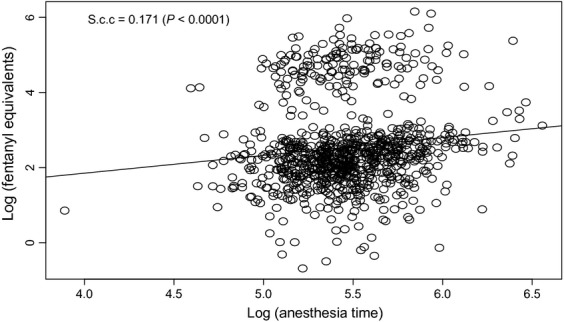
Correlation between anethesia time and fentalyl equivalent use. The figure demonstrates a statistically significant correlation between the time that the patients spent under anesthesia and the use of fentanyl equivalents during surgery. The scatter plot below showed that the Spearman correlation coefficient was significantly different from 0.

The median amount of fentanyl equivalents administered intraoperatively was 10.15 (6.37–16.47) *μ*g/kg and this number was subsequently utilized to identify the dose separating low and high intraoperative doses of fentanyl. The mean duration was surgery was 233 (193––286) min. The distribution of fentanyl equivalents and duration of surgery as demonstrated by the BLiP plot were not normal, thus we performed logarithmic transformations to approximate to normal distributions (Fig.2). As the amount of opioids administered intraoperatively could be directly related to the duration of surgery, we assessed the correlation between these two variables. As shown in Figure3, we demonstrated a significant correlation between the amount of fentanyl equivalents administered intraoperatively and the duration of surgery.

## RFS in the overall population

The median RFS time was 67.54 months and the rate of RFS at 3 and 5 years was 63.6% and 53%, respectively (Table2). Table3A shows the risk factors associated with better RFS. The intraoperative opioid use of fentanyl equivalents was not associated with significant changes in RFS (HR: 1.074, 95% CI: 0.98–1.16, *P* = 0.08). As the decision of using the median of fentanyl use as a cutoff point was arbitrary, we reanalyzed the fentanyl equivalent consumption as a continuos variable. The multivariate analysis supported our initial finding that the amount of fentanyl equivalents given intraoperatively was not associated with a change in RFS (HR: 1.074, 95% CI: 0.98–1.11, *P* = 0.088). In a second model of multivariate analysis, we included surgical times as a covariate because time and the amount of fentanyl equivalents give intraoperatively were correlated. The analysis showed that a longer surgical time was a predictor factor for RFS (HR: 1.501, 95% CI: 1.05–2.12, *P* = 0.022). The probability of RFS by stage is illustrated using a Kaplan–Meier graph in Figure3.

**Table 2:** RFS rates at years 3 and 5 for the overall population of patients with NSCLC.

Variable	Level	*N*	Event	Median RFS in months (95% CI)	RFS at 3 years (95% CI)	RFS at 5 years (95% CI)	*P*-value
Age	<65	393	150	81.7 (77.43, NA)	0.68 (0.64, 0.73)	0.61 (0.56, 0.66)	0.0001
	≥65	505	265	53.48 (41.2, 66.69)	0.58 (0.54, 0.63)	0.47 (0.43, 0.52)	
BMI	≤25	330	174	46.52 (33.38, 64.85)	0.55 (0.49, 0.6)	0.44 (0.39, 0.51)	0.0024
	>25	568	241	74.24 (67.54, 87.52)	0.67 (0.63, 0.71)	0.58 (0.54, 0.62)	
Gender	F	423	175	81.7 (71.09, NA)	0.67 (0.63, 0.72)	0.59 (0.55, 0.64)	0.0003
	M	475	240	53.48 (41.85, 67.97)	0.58 (0.54, 0.63)	0.47 (0.42, 0.52)	
ASA	2	85	32	77.63 (63.24, NA)	0.69 (0.59, 0.8)	0.61 (0.51, 0.73)	0.0128
	3	763	349	67.97 (58.41, 81.7)	0.62 (0.59, 0.66)	0.53 (0.5, 0.57)	
	4	48	33	38.81 (20.4, 68.79)	0.54 (0.42, 0.7)	0.36 (0.25, 0.53)	
Stage	1	485	168	87.19 (81.8, NA)	0.74 (0.7, 0.78)	0.64 (0.6, 0.69)	<0.0001
	2	186	104	50.39 (31.11, 66.52)	0.56 (0.49, 0.63)	0.45 (0.38, 0.53)	
	3	225	143	20.27 (14.72, 33.08)	0.43 (0.37, 0.5)	0.35 (0.29, 0.42)	
Fentanyl (*μ*g/kg)	<10.15	447	185	77.43 (65.54, NA)	0.67 (0.63, 0.72)	0.57 (0.52, 0.62)	0.0093
	≥10.15	451	230	55.22 (43.17, 71.09)	0.58 (0.54, 0.63)	0.49 (0.44, 0.54)	
Preoperative chemotherapy	N	714	300	77.63 (67.97, NA)	0.67 (0.64, 0.71)	0.58 (0.54, 0.62)	<0.0001
	Y	183	115	20.01 (14.06, 40.47)	0.44 (0.37, 0.52)	0.34 (0.28, 0.43)	
Preoperative radiation	N	864	392	68.79 (62.29, 81.7)	0.63 (0.6, 0.67)	0.54 (0.51, 0.58)	<0.0001
	Y	34	23	14.06 (7.1, 54.99)	0.38 (0.24, 0.59)	0.23 (0.1, 0.49)	
Postoperative chemotherapy	N	622	276	68.66 (58.51, NA)	0.63 (0.6, 0.67)	0.54 (0.5, 0.58)	0.2293
	Y	275	139	60.35 (44.51, 81.7)	0.6 (0.55, 0.67)	0.5 (0.45, 0.57)	
Postoperative radiation	N	760	323	77.43 (67.05, NA)	0.66 (0.63, 0.7)	0.57 (0.53, 0.6)	<0.0001
	Y	136	91	21.42 (17.35, 45.3)	0.44 (0.36, 0.53)	0.33 (0.26, 0.43)	
Thoracotomy	N	189	75	77.63 (64.85, NA)	0.68 (0.62, 0.75)	0.59 (0.52, 0.67)	0.0805
	Y	708	339	66.52 (51.35, 75.33)	0.61 (0.58, 0.65)	0.51 (0.48, 0.55)	
Thoracoscopy	N	756	369	60.35 (50.39, 72.34)	0.6 (0.56, 0.64)	0.5 (0.47, 0.54)	0.0003
	Y	141	45	NA (69.97, NA)	0.77 (0.7, 0.84)	0.68 (0.6, 0.77)	

**Table 3:** Multivariate Cox proportional hazard model for RFS in the overall population of (A) patients with (B) stage I, (C) stage II, and (D) stage III.

Variable	*P*-value	HR	95% CI	
(A) Overall population of patients				
Age	<0.0001	1.035	1.023	1.046
BMI	0.0482	0.979	0.959	1.000
Gender				
F vs. M	0.0105	0.770	0.631	0.941
ASA				
2 vs. 4	0.0229	0.553	0.332	0.921
3 vs. 4	0.0017	0.550	0.379	0.799
Stage				
I vs. III	<0.0001	0.510	0.390	0.667
II vs. III	0.2029	0.837	0.637	1.100
Fentanyl	0.0887	1.074	0.989	1.166
Preoperative chemotherapy				
N vs. Y	0.0003	0.641	0.506	0.814
Postoperative radiation				
N vs. Y	0.0387	0.753	0.576	0.985
Thoracoscopy				
N vs. Y	0.0935	1.317	0.955	1.816
(B) Stage I				
Age	<0.0001	1.039	1.022	1.056
BMI	0.0010	0.942	0.910	0.976
Gender				
F vs. M	0.0004	0.561	0.409	0.771
Fentanyl	0.0531	1.127	0.998	1.272
Preoperative chemotherapy				
N vs. Y	0.0333	0.596	0.370	0.960
Postoperative radiation				
N vs. Y	0.0623	0.540	0.283	1.032
(C) Stage II				
Age	<0.0001	1.044	1.022	1.067
Fentanyl	0.8331	1.019	0.854	1.216
Preoperative radiation				
N vs. Y	0.0755	0.400	0.145	1.099
(D) Stage III				
Preoperative chemotherapy				
N vs. Y	0.0233	0.680	0.487	0.949
Postoperative chemotherapy				
N vs. Y	0.0487	1.396	1.002	1.946
Fentanyl	0.8723	1.012	0.876	1.169

The median RFS for stage I patients was 87.19 months and the RFS rates at 3 and 5 years were 74% and 64%, respectively. One patient with missing information on follow-up for recurrence evaluation was excluded from the analysis. As shown in Table3, the multivariate analysis demonstrated that age 65 years or younger and female gender were associated with longer RFS, and the administration of preoperative or postoperative chemotherapy and radiation was associated with poorer RFS. When analyzed as continuous variable, the multivariate analysis demonstrated that a trend toward better RFS if the requirements of fentanyl equivalents were low intraoperatively (HR: 1.127, 95% CI: 0.99–1.12, *P* = 0.053) (Table3B). Importantly, we did no observe any association between surgical time and RFS (HR: 1.091, 95% CI: 0.60–1.96, *P* = 0.769).

The median RFS for patients with stage II was 50.39 months and the RFS rates at 3 and 5 years were 56% and 45%, respectively. One patient with missing information on follow-up for recurrence evaluation was excluded from the analysis. The multivariate analysis demonstrated that age 65 years or younger was the only variable associated with favorable RFS. The analysis also indicated that the amount of fentanyl equivalents administered intraoperatively had no effect on RFS (HR: 1.019, 95% CI: 0.85–1.26, *P* = 0.833) (Table3C). We found no association between duration of surgery and RFS when that covariate was included in the statistical model (HR: 1.1.853, 95% CI: 0.91–3.752, *P* = 0.086).

The median RFS for stage III patients was the shortest among all stages (20.17 months) and the RFS rates at 3 and 5 years were the lowest among the three stages of disease (Table3D). One patient with missing information on follow-up for recurrence evaluation was excluded from the analysis. The univariate and multivariate analysis demonstrated that the only variable associated with longer RFS was the administration of chemotherapy postoperatively. In contrast, use of preoperative chemotherapy was associated with a worse RFS. Fentanyl equivalents administered intraoperatively had no effect on RFS (HR: 1.012, 95% CI: 0.87–1.16, *P* = 0.872) (Table3D). When we included duration of surgery in the statistical model, we found a trend toward an association between the duration of surgery and RFS (HR: 1.6.37, 95% CI: 0.97–2.75, *P* = 0.063).

## Overall survival

The median OS time for the entire population was 82.39 months (Table4). In the multivariate analysis, variables that were associated with better survival were age younger than 65 years, female gender, and thoracoscopy. American physical status of 4, stage III, preoperative chemotherapy and radiation, and open thoracotomy were all predictors of poor OS. Importantly, when we included in the multivariate analysis time of surgery or fentanyl equivalents as a continuous variable, they were not associated with a change in OS (HR: 1.986, 95% CI: 0.96–1.16, *P* = 0.231 and HR: 1.34, 95% CI: 0.90–1.99, *P* = 0.14).

**Table 4:** OS rates for the overall population of patients.

Variable	Level	*N*	Event	Median OS in months (95% CI)	OS at 3 years (95% CI)	OS at 5 years (95% CI)	*P*-value
Age	<65	394	107	NA (NA, NA)	0.8 (0.76, 0.84)	0.71 (0.66, 0.76)	<0.0001
	≥65	507	214	75.53 (68.66, NA)	0.69 (0.65, 0.73)	0.58 (0.53, 0.62)	
BMI	≤25	331	135	82.39 (77.63, NA)	0.69 (0.64, 0.74)	0.57 (0.51, 0.63)	0.0124
	>25	570	186	NA (78.65, NA)	0.76 (0.73, 0.8)	0.68 (0.64, 0.72)	
Gender	F	425	127	NA (NA, NA)	0.79 (0.75, 0.83)	0.7 (0.65, 0.74)	<0.0001
	M	476	194	75.53 (68.66, NA)	0.68 (0.64, 0.73)	0.58 (0.53, 0.63)	
ASA	2	86	23	NA (77.63, NA)	0.81 (0.73, 0.9)	0.71 (0.62, 0.83)	0.0263
	3	764	272	NA (81.7, NA)	0.73 (0.7, 0.77)	0.63 (0.6, 0.67)	
	4	49	26	61.17 (35.74, NA)	0.61 (0.49, 0.77)	0.53 (0.4, 0.69)	
Stage	1	486	123	NA (NA, NA)	0.84 (0.8, 0.87)	0.74 (0.7, 0.79)	<0.0001
	2	187	78	77.63 (65.97, NA)	0.69 (0.62, 0.76)	0.59 (0.52, 0.67)	
	3	226	120	45.99 (33.9, 81.7)	0.56 (0.49, 0.63)	0.44 (0.38, 0.51)	
Fentanyl (*μ*g/kg)	<10.15	450	146	82.39 (78.65, NA)	0.76 (0.72, 0.8)	0.67 (0.62, 0.72)	0.0993
	≥10.15	451	175	NA (75.33, NA)	0.71 (0.67, 0.76)	0.6 (0.56, 0.65)	
Preoperative chemotherapy	N	715	231	NA (81.8, NA)	0.77 (0.74, 0.8)	0.67 (0.64, 0.71)	<0.0001
	Y	185	90	54.99 (44.02, NA)	0.61 (0.54, 0.68)	0.49 (0.42, 0.57)	
Preoperative	N	867	300	NA (79.24, NA)	0.75 (0.72, 0.78)	0.65 (0.61, 0.68)	<0.0001
Radiation	Y	34	21	17.94 (13.96, NA)	0.44 (0.3, 0.65)	0.3 (0.16, 0.55)	
Postoperative	N	623	220	NA (77.43, NA)	0.73 (0.7, 0.77)	0.64 (0.6, 0.68)	0.7713
Chemotherapy	Y	277	101	81.8 (79.24, NA)	0.74 (0.69, 0.8)	0.63 (0.57, 0.7)	
Postoperative radiation	N	763	241	NA (82.39, NA)	0.77 (0.74, 0.8)	0.68 (0.65, 0.72)	<0.0001
	Y	136	79	48.69 (34.86, 59.92)	0.57 (0.49, 0.66)	0.39 (0.31, 0.49)	
Thoracoscopy	N	759	288	81.7 (77.43, NA)	0.71 (0.68, 0.75)	0.61 (0.58, 0.65)	0.0009
	Y	141	32	NA (NA, NA)	0.85 (0.8, 0.92)	0.76 (0.69, 0.84)	

The OS in stage I patients at 3 and 5 years the OS rate was 84% and 74%, respectively. After adjusting for important covariates, the multivariate analysis showed a significant association between those same variables and longer OS. Remarkably, the amount of fentanyl equivalents used intraoperatively was significantly associated with a better OS (HR: 1.15, 95% CI: 1.01–1.32, *P* = 0.036) (Table5). Even when we found a direct correlation between the amounts of fentanyl administered intraoperatively and the duration of surgery, we did not identify this last covariate as a risk factor for OS (HR: 0.82, 95% CI: 1.42–1.59, *P* = 0.573).

**Table 5:** Multivariate Cox proportional hazard model for OS in the overall population of patients with (A) NSCLC, (B) stage I, (C) stage II, and (D) stage III.

Analysis of maximum likelihood estimates				
Variable	*P*-value	HR	95% CI	
(A) Overall population of patients with NSCLC				
Age	<0.0001	1.45	1.032	1.058
Body mass index	0.0500	0.977	0.955	1.000
Gender				
F vs. M	0.0035	0.709	0.563	0.893
ASA				
2 vs. 4	0.0680	0.577	0.320	1.042
3 vs. 4	0.0163	0.598	0.392	0.910
Stage				
I vs. III	<0.0001	0.481	0.358	0.648
II vs. III	0.0495	0.736	0.543	0.999
Fentanyl equivalents	0.2318	1.060	0.964	1.165
Preoperative radiation				
N vs. Y	0.0078	0.534	0.337	0.848
Postoperative radiation				
N vs. Y	0.0025	0.639	0.477	0.854
Thoracoscopy				
N vs. Y	0.0477	1.463	1.004	2.132
(B) Stage I				
Age	<0.0001	1.051	1.029	1.073
Body mass index	0.0026	0.939	0.902	0.978
Gender				
F vs. M	<0.0001	0.422	0.286	0.622
ASA				
2 vs. 4	0.0127	0.237	0.076	0.735
3 vs. 4	0.0216	0.508	0.285	0.906
Fentanyl equivalents	0.0360	1.158	1.010	1.329
Postoperative radiation				
N vs. Y	0.0156	0.428	0.215	0.851
(C) Stage II				
Age	<0.0001	1.057	1.030	1.084
Fentanyl equivalents	0.5869	0.942	0.761	1.167
Preoperative radiation				
N vs. Y	0.0016	0.191	0.068	0.535
(D) Stage III				
Age	0.0442	1.022	1.001	1.043
LogFentanyl	0.8621	0.986	0.839	1.158
Preoperative radiation				
N vs. Y	0.1439	0.661	0.379	1.152
Postoperative chemotherapy				
N vs. Y	0.0315	1.506	1.037	2.188

The median OS time in patients with stage II and III was 77.63 months and 45.99 months, respectively. Our univariate and multivariate analysis demonstrated that age less than 65 years was associated a better OS in both groups of patients. In patients with stage II, preoperative radiation was associated with a poor OS in the univariate and multivariate analysis (Table5), in contrast the administration of postoperative chemotherapy was associated with better survival in patients with stage III (Table5). The multivariate analysis demonstrated no significant association between amount of fentanyl equivalents administered intraoperatively and longer OS for neither stage II (HR: 0.94, 95% CI: 0.76–1.16, *P* = 0.586) nor stage III (HR: 0.98, 95% CI: 0.83–1.15, *P* = 0.862) (Table5). When we included duration of surgery into the statistical model, we found no association between this covariate and OS neither for patients with stage II or III HR: 2.105, 95% CI: 0.92–4.77, *P* = 0.074 and HR: 1.56, 95% CI: 0.86–2.82, *P* = 0.137, respectively), although there was trend toward better OS in patients with stage II that had shorter surgical times.

## Discussion

Researchers have tried to answer the question of whether perioperative interventions such as surgery itself, anesthetics and analgesics have an impact on cancer outcomes. Although, it is suggested that the use of opioid-based anesthetic techniques would be associated with unfavorable oncological outcomes (RFS), the results from recent studies are conflicting 1,12–15. In fact, we have recently demonstrate no association between the type of postoperative analgesia used after NSCLC and longer RFS or OS; however, in that study, we did not quantify the amount of opioids used postoperatively 16. Two recent clinical studies by Forget et al. and Zylla et al. have highlighted the importance of opioids in the context of cancer outcomes. The study by Forget et al. suggested that the use of sufentanil was associated with shorter biochemical-free recurrence survival in patients with prostate cancer and Zylla et al. demonstrated that large opioid requirement and high expression of the MOR were associated with shorter progression-free survival and OS in patients with metastatic prostate cancer 17,18 To complicate the matter, the association between the intraoperative administration of sufentanil and higher rate of recurrence after breast cancer surgery was not significant 19. Evidence for a human study also indicates that MORs are expressed in NSCLC tumors; thus it is possible to think that the use of opioids might modulate the proliferative and invasive features of NSCLCs 20.

Stage of the disease for NSCLC is known to be a significant determinant of outcomes related to cancer recurrence and mortality in patients with lung cancer. We, therefore, performed a subanalysis for each stage of the disease to investigate the relationship between RFS and OS, and the variables collected in our patients 21. Interestingly, we did not find an association between fentanyl equivalents and RFS in patients with stage II and III. However, an important favorable trend (HR: 1.12, CI 95% 0.99–1.27, *P* = 0.053) was observed in patients with stage I of the disease. This trend would suggest that a reduced intraoperative administration of opioids in patients with early stage of the disease might be associated with a longer RFS. Remarkably, a similar association was found for OS in patients with stage I disease. Lennon et al. have demonstrated that most cell lines for NSCLC express MOR and its overexpression has been associated with increased migrative and invasive features 22. They also showed that morphine in vitro*,* increased NSCLC cell proliferation, and the blockade of the MOR receptor-reduced metastasis formation in animals bearing lung cancer 3.

Our data suggest that the influence of opioids on cancer recurrence shows an important trend in the early (stage I) rather than later (stage II and III) stages. Our findings might be explained by several hypotheses. It is possible to speculate that in later stages of the disease, tumor growth may be influenced by mechanisms other than by MOR activation, including the influence of other opioids receptors such as *δ*-opioid receptors. In fact, the receptor binding is much higher for *δ*- than MOR in lung cancer cells 23. Moreover, long-term exposure to opioids appears to favor a protumoral microenvironment 24. As patients with higher stages are more likely to have longer, complex, and more invasive surgeries, it could be argued that our results reflect the association of higher intraoperative consumption/administration of opioids to patients undergoing complex and longer procedures. The number of mutations increases exponentially as tumors grow, hence the mechanisms driving cell survival or apoptosis might change in different stages of the disease 25. Epidermal growth factor receptor (EGFR) mutations can occur at different rates in patients with primary tumors, those with regional recurrences, and distant metastasis 26. Interestingly, opioids can induce phosphorylation of the EGFR; hence, the growth of cancer cells expressing EGFR and MOR at different rates in the so-called postoperative minimal residual disease (MRD; circulating tumor cells, micrometastasis, and positive surgical margins) could be modulated differently by the different opioids 27.

Another explanation could be linked to the immune-suppressive effect of opioids. Our group has recently demonstrated that immediately after lung cancer surgery there is a profound depression of the innate immunity, which is characterized by a decrease in the function of the natural killer cells and it has been demonstrated that opioids in moderate and large amounts depress their function 28,29. Based on these premises, we can hypothesize that patients with no or “limited” MRD (Stage I) who received low amount of opioids appear to have an improved survival because the impact of the postoperative immune suppression is less important compared to that in those with significantly more MRD (stage II/III), who may have received higher doses of opioids due to more complex and invasive surgical procedures. Although, this last explanation is merely speculative, similar results have been found with other perioperative interventions such as blood transfusions, which have immune-suppressive effects 30.

Our study has several limitations. First, this is a retrospective study hence there are unknown factors that may have affected the studied outcomes. Second, we included patients with different histologies. The expression of *μ* and other opioid receptors may therefore be different, hence the varied effects of opioids on the tumor biology and disease progression. Third, we did not adjust for type of pre- and postoperative chemotherapy regimen in our patient population. Some patients in our database might have been treated with these newer agents (monoclonal antibodies against growth factors, and tyrosine kinase inhibitors) based on their tumor mutation profile. This is important to consider because of the well-described interaction (coactivation of mu-receptors and VEGF with opioid agonists) between the mu receptor and growth factor receptors 27. Fourth, although we arbitrarily defined fentanyl equivalents in the univariate analysis, in the multivariate analysis opioids use was considered as a continuous variable. Fourth, it is important to remark in our analysis several risk factor such as age, gender, stage, and adjuvant treatment also showed an association with RFS and OS, thus suggesting that any of these factors are perhaps more relevant that the use of intraoperative opioids. Finally, we did not account for the amount of opioids administered postoperatively and long-term after surgery. This is important because some of the protumoral effects of opioids are observed after chronic exposure 24. Although we were not able to reliably collect the total amount of opioids administered in the postoperative period and beyond, it is important to remark that we recently reported that the type of postoperative analgesia appear not to be risk factor for RFS and OS in a similar but smaller population of patients 16.

In conclusion, our results indicate an association between the amounts of opioid administered during surgery and OS only after lung cancer resection for stage I disease. Until our findings are confirmed in a randomized controlled trial, opioids will continue to play a key part in the operative management of analgesia needs during major cancer surgery.

## Conflict of Interest

None declared.
